# Sexually transmitted infections in oral cavity lesions: Human papillomavirus, *Chlamydia trachomatis*, and Herpes simplex virus

**DOI:** 10.1080/20002297.2019.1632129

**Published:** 2019-06-25

**Authors:** Jessica P. Mosmann, Angel D. Talavera, María I. Criscuolo, Raúl F. Venezuela, Ana X. Kiguen, Rene Panico, Ruth Ferreyra De Prato, Silvia A. López De Blanc, Viviana ré, Cecilia G. Cuffini

**Affiliations:** a Instituto de Virología “Dr. J. M. Vanella”, Facultad de Ciencias Médicas – Universidad Nacional de Córdoba, Córdoba, Argentina; b Consejo Nacional de Investigaciones Científicas y Técnicas (CONICET), Córdoba, Argentina; c Cátedra de Estomatología, Facultad de Odontología- Universidad Nacional de Córdoba, Córdoba, Argentina

**Keywords:** Oral cavity, Human papillomavirus, Chlamydia trachomatis, Herpes simplex virus, sexually transmitted infections, benign lesions, potentially malignant disorders, oral squamous cell carcinoma

## Abstract

**Objective**: Provide evidence of HPV, *C. trachomatis,* and HSV infection in the oral cavity from patients with different types of stomatological lesions.

**Materials and Methods**: Oral swabs samples were collected from a total of 318 patients. The infectious agents were analyzed using the PCR technique. HPV genotyping and HSV type were studied using the RFLP method.

**Results**: We studied 137 benign lesions (B), 96 potentially malignant disorders (PMD) and 85 oral squamous cell carcinomas (OSCC). The prevalence of HPV was 34%. The most frequently genotypes detected were 6 low risk and 16 high risk. The prevalence of *C. trachomatis* was 16% and HSV 3%. Co-infections were detected mostly in benign lesions as following: HPV-*C. trachomatis* in 4%, *C. trachomatis*- HSV in 1.8% and HPV-HSV in 0.3%.

**Conclusion**: This report is the first contribution to the identification and genotype characterization of HPV in a scenario little studied in our area, and it also contributes to improving our understanding on sexually transmitted infectious agents and their associations with the oral cavity. Besides, we detect the presence of *C. trachomatis* and HSV and co-infection with HPV in the oral cavity, which they should be taken into account for diagnostic and treatment purposes.

## Introduction

Human Papillomavirus (HPV) is one of the most frequent sexually transmitted infections (STIs) that could play a role in the pathogenesis of head and neck cancer. This infection is a major health problem worldwide that manifests as progressive squamous dysplasia and results from cumulative genetic and epigenetic alterations induced by exposure to carcinogenic agents [1,2].

Despite the well-established role of HPV in cervical cancer, new lines of evidence suggest that HPV may also be an independent risk factor for oral cancer [3], and co-infection with other STI could increase the risk for cancer. Many factors are associated with HPV infection, such as tobacco, alcohol, steroid hormones, UV, bacterial and viral infections [4].

There is evidence that *Chlamydia trachomatis (C. trachomatis)* infection could act as a co-factor which facilitates HPV infection and contributes to the viral persistence, increasing the risk of developing cervical neoplasia [5]. *C. trachomatis* is a gram-negative obligate intracellular bacterium with a biphasic development that often remains asymptomatic and can infect genital and oral mucosa [6,7]. In a meta-analysis study, Zhu et al. [8] reported that individuals infected with *C. trachomatis* have a higher risk of developing cervical cancer.

On the other hand, Herpes simplex virus (HSV) in conjunction with HPV infection may increase the risk of cervical cancer [4]. HSV is an epitheliotropic human pathogen which establishes latency in the sensory ganglia, and it can be reactivated in response to stress, causing secondary infection in oral epithelial cells. Just like HPV infection, published data demonstrates that HSV co-infection with *C. trachomatis* could induce chlamydial persistence [9,10].

The aim of this work was to provide evidence of HPV, *C. trachomatis* and HSV infection in oral cavity samples collected from patients from the central region of Argentina with different types of stomatological lesions.

## Materials and methods

### Study population and sampling

Oral swabs samples from a total of 318 patients over 18 years were clinically evaluated in the Department of Stomatology–Faculty of Dentistry–National University of Córdoba–Argentina. Samples were obtained of oral lesions which were classified by specialized stomatologist and confirmed by histopathological study, according to three types: benign lesions (B), potentially malignant disorders (PMDs) and oral squamous cell carcinoma (OSCC).

### Ethical approval

This study was approved by the Institutional Committee of Ethics of Health ‘Oulton Romagosa’ (RePIS 007) of Córdoba- Argentina, according to the ethical principles stated in the declaration of Helsinki.

#### DNA extraction and HPV, *C. trachomatis*, HSV detection

Viral and bacterial DNA was extracted using the commercial AccuPrepGenomic DNA Extraction Kit-Bioneer, following the manufacturer’s instructions. Then, HPV L1 genomic region (450 bp) was amplified with degenerate primers MY09 and MY11 following the protocol previously described by Manos et al. [11]. HPV-DNA positive samples were typed by restriction fragment length polymorphism (RFLP) method, using seven different restriction enzymes (BamHI, DdeI, HaeIII, HinfI, PstI, RsaI, and Sau3AIII) [12]. *C.trachomatis* was detected using CTP1 and CTP2 primers to cryptic plasmid obtained amplicons size of 201 bp [13]. We used a PCR with B3/B4 primer set designed to amplify a genomic fragment of highly conserved and divergent DNA sequences in the gene encoding the glycoprotein B (Gb) of primate α-herpesvirus. Then, RFLP with HaeIII restriction enzyme was used to differential identification of HSV-1 (555 bp) and HSV −2 (561 bp) [14].

The PCR products were visualized by electrophoresis in 1.5% agarose gel using a UV transilluminator. The β-globin gene was used as a DNA preservation marker.

#### Statistical analysis

Results were analyzed using Chi-square (X^2^) and Fisher Exact with a significance level 5% (95% CI), Epi info™ CDC software.

## Results

**Figure 1.**
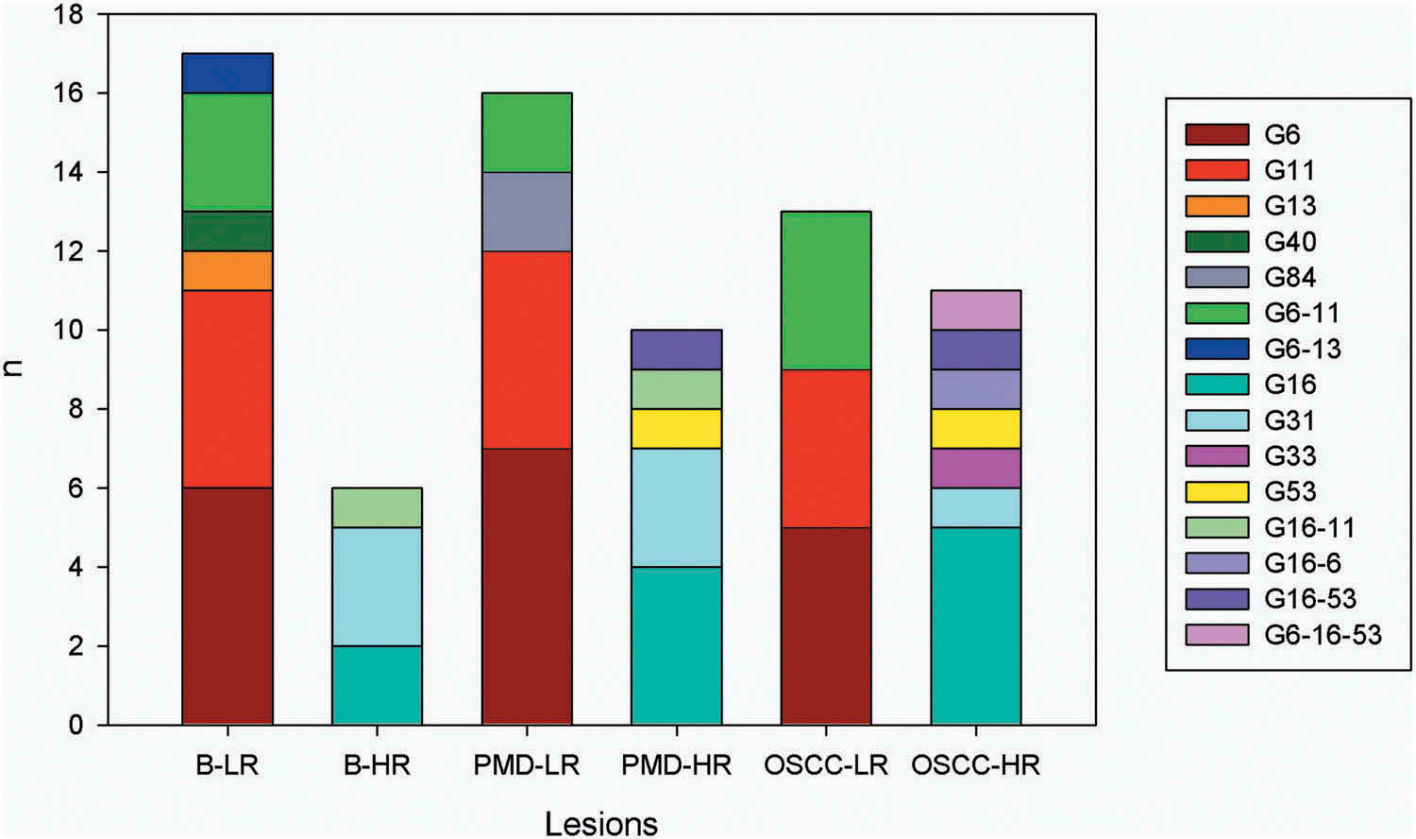
HPV genotypes according to lesions. **G**: genotype**B-LR**: benign lesion-low risk genotype, **B-HR**: benign lesion-high risk genotype, **PMD-LR**: potentially malignant disorder-low risk genotype, **PMD-HR**: potentially malignant disorder-high risk genotype, **OSCC-LR**: oral squamous cell carcinoma-low risk genotype, **OSCC-HR**: oral squamous cell carcinoma-high risk genotype.

We evaluated a total of 318 patients, 169 females, and 149 males, aged between 20 and 88 years (mean 55). Of these, 137 were classified among benign lesions (B), 96 potentially malignant disorders (PMDs) and 85 oral squamous cell carcinomas (OSCC). Positivity of DNA-HPV was observed in 34.3% (109/318) (Table 1). It was possible to identify HPV genotypes in 66.9% (73/109): that were showed low risk (LR) genotypes (6, 11, 13, 40, 84) in 63.0% (46/73) of the cases and high risk (HR) genotypes (16, 31, 33) in 37% (27/73). In 22% (16/73) of the cases, LR-HR co-infections were detected (Table 2). According to the category of the lesion, HR genotypes detection increased while the progression of the injury was worse. Thus, HR genotype was detected in 26.1%, 38.5%, and 46.8% of B, PMDs, and OSCC, respectively. Globally, the most frequent genotypes detected were 6 (LR) and 16 (HR). But genotypes LR: 11, 13, 40, 84 and HR: 31, 33 and 53 (intermediate risk) were detected. It is important to remark that genotype 53 was detected only in PMDs and OSCC lesions and co-infections with genotype 16 (Figure 1).

**Table 1.** Percentage of STI according to sex and lesion.

		Total		HPV DNA positive					*C. trachomatis* DNA positive					HSV DNA positive				
		N = 318 (100%)		n = 109 (34%)					n = 54 (17%)					n = 10 (3%)				
		n	(%)	n	(%)	p value	OR	IC (95%)	n	(%)	p value	OR	IC (95%)	n	(%)	p value	OR	IC (95%)
Sex	**Male**	149	46,9	50	45.9	0.89	1.06	(0.66–1.68)	27	50	0.71	0.85	(0.47–1.54)	1	10	0.02	1.73	(1.37–2.18)
	**Female**	169	53,1	59	54.1				27	50				9	90			
Lesion	**B**	137	43.1	39	35.8	0.07	1.58	(0.98–2.55)	27	50	0.32	0.71	(0.39–1.28)	8	80	0.02	0.34	(0.090–1.19)
	**PMD**	96	30.2	34	31.2				18	33.3				1	10			
	**OSCC**	85	26.7	36	33				9	16.7				1	10			

**B**: benign lesion, **PMD**: potentially malignant disorders; **OSCC**: oral squamous cell carcinoma.

**Table 2.** Characteristics of HPV genotyped samples (n = 73).

SEX	LESION	HPV GENOTYPE	GENOTYPE RISK	*C. trachomatis*	HSV
F	B	6	LR	-	-
M	B	6 and 11	LR	-	-
M	B	11	LR	-	-
M	B	11	LR	-	-
M	B	6	LR	-	-
M	B	6 and 11	LR	**+**	-
M	B	11	LR	-	-
F	B	11	LR	-	-
M	B	6 and 11	LR	-	-
M	B	6	LR	-	-
M	B	40	LR	-	-
F	B	13	LR	**+**	-
F	B	6	LR	-	-
F	B	6 and 13	LR	**+**	**+**
F	B	6	LR	**+**	-
M	B	11	LR	-	-
M	B	6	LR	-	-
F	B	16	HR	-	-
F	B	31	HR	-	-
F	B	31	HR	-	-
F	B	16	HR	-	-
F	B	31	HR	-	-
F	B	16 and 11	HR	**+**	-
M	PMD	6 and 11	LR	-	-
F	PMD	11	LR	-	-
F	PMD	6	LR	-	-
M	PMD	6 and 11	LR	-	-
F	PMD	6	LR	-	-
M	PMD	6	LR	-	-
F	PMD	6	LR	-	-
F	PMD	6	LR	-	-
F	PMD	84	LR	-	-
M	PMD	6	LR	-	-
M	PMD	11	LR	-	-
M	PMD	11	LR	-	-
F	PMD	11	LR	**+**	-
F	PMD	11	LR	-	-
M	PMD	6	LR	-	-
F	PMD	84	LR	**+**	-
F	PMD	16 and 53	HR	-	-
F	PMD	31	HR	-	-
F	PMD	53	HR	-	-
F	PMD	16	HR	-	-
M	PMD	11 and 16	HR	**+**	-
F	PMD	16	HR	-	-
F	PMD	16	HR	-	-
F	PMD	16	HR	-	-
F	PMD	31	HR	-	-
F	PMD	31	HR	-	-
M	OSCC	11	LR	-	-
M	OSCC	11 and 6	LR	-	-
M	OSCC	6 and 11	LR	-	-
M	OSCC	6	LR	-	-
M	OSCC	11	LR	-	-
M	OSCC	6	LR	**+**	-
M	OSCC	6	LR	**+**	-
M	OSCC	11	LR	**+**	-
M	OSCC	6	LR	-	-
F	OSCC	11	LR	-	-
M	OSCC	6 and 11	LR	-	-
F	OSCC	6	LR	-	-
M	OSCC	11 and 6	LR	-	-
M	OSCC	16	HR	-	-
M	OSCC	6 and 16	HR	-	-
F	OSCC	33	HR	-	-
M	OSCC	16	HR	-	-
F	OSCC	16	HR	-	-
F	OSCC	16	HR	-	-
F	OSCC	16 and 53	HR	-	-
M	OSCC	31	HR	-	-
F	OSCC	53	HR	-	-
M	OSCC	16	HR	-	-
F	OSCC	6, 16 and 53	HR	-	-

**B**: benign lesion, **PMD**: potentially malignant disorders; **OSCC**: oral squamous cell carcinoma.

**HR**: high risk; **LR**: low risk

The prevalence of *C. trachomatis* was 16.9% (54/318) and for HSV was 3% (10/318) (Table 1). HSV 1 and 2 were detected in 7 and 3 samples, respectively. Out of 10 HSV positive, 8 samples were B lesion and 6 were *C. trachomatis* co-infection (Table 3).

**Table 3.** Characteristics of HSV positive patients (n = 10).

HSV TYPE	SEX	LESION	HPV/GENOTYPE/RISK	*C. trachomatis*
1	F	B	-	**+**
1	F	B	-	**+**
1	F	B	-	-
1	F	B	-	-
1	F	B	-	**+**
*2	F	B	**+/6–13/LR**	**+**
2	F	B	-	-
2	F	B	-	-
1	F	PMD	-	**+**
1	M	OSCC	-	**+**

**B**: benign lesion, **PMD**: potentially malignant disorders; **OSCC**: oral squamous cell carcinoma.

**HR**: high risk; **LR**: low risk.

*Female patient with triple infection (HPV, *C. trachomatis* and HSV.)

There were no statistically significant differences in DNA of HPV and *C. trachomatis* detection by sex, age and type of oral lesion (p > 0.05), although the presence of HSV was significantly associated to sex and type of lesion variables (p = 0.02) (Table 1).

Globally, more frequent co-infection was HPV- *C. trachomatis* (4%), followed by *C. trachomatis*- HSV (1.8%), finally HPV-HSV co-infection was 0.3%. In most of the co-infections, HPV LR genotypes were detected (Table 4). One female patient had a triple infection in a benign lesion with LR HPV genotypes.

**Table 4.** STI co-infections in oral samples from 318 Argentinean patients.

		Total		Co infection HPV-*C. trachomatis*		Co infection HPV-HSV		Co infection (*C. trachomatis*-HSV)	
		*N* = 318 (100%)		*N* = 13 (4%)		*N* = 1 (0.3%)		*N* = 6 (1.8%)	
		*n*	(%)	*n*	(%)	*n*	(%)	*n*	(%)
Sex	**Male**	149	46.9	6	46.2	0	0	1	16.7
	**Female**	169	53.1	7	53.8	1	100	5	83.3
Lesion	**B**	137	43.1	6	46.1	1	100	4	66.6
	**PMD**	96	30.2	4	30.8	0	0	1	16.7
	**OSCC**	85	26.7	3	23.1	0	0	1	16.7
HPV	**HR**			2	15.4	0	0		
genotype	**LR**			9	69.2	1	100		
risk	**W/G**			2	15.4	0	0		

**B**: benign lesion, **PMD**: potentially malignant disorders; **OSCC**: oral squamous cell carcinoma.

**HR**: high risk; **LR**: low risk; **W/G**: without genotype detected.

## Discussion

Despite the fact that oral transmission of HPV is unclear, different sexual habits, such as the onset of sexual activity at an earlier age, an increase in the number of partners, and orogenital sex have made HPV an endemic infection [2]. As a result, some studies have shown the role of viruses in head and neck cancer [15–17].

Since the histology of oral mucosa resembles that of the uterine cervix, we can anticipate the presence of HPV and other STI which are detected in different lesions of genital areas and the oral mucosa. It is estimated that most of the adults who are sexually active, have been exposed to HPV at any anatomical site [18].

Co-infection with two or more pathogens may be an important cofactor for initiation and/or progression of the oncogenic transformation of epithelial cells of the oral mucosa [17]. Several infectious agents may cause a latent infection, and it is well known that pathogenic infections are necessary, but not sufficient for cancer [19].

HPV was reported in several benign oral lesions, especially verruca vulgaris, squamous papilloma, and the rare focal epithelial hyperplasia [2]. Malignant transformation, OSCC, presents different clinical aspects related to the location of the tumor, precancerous lesions and risk factors [20].

In the present study, the prevalence of HPV in oral cavity lesions was 34%. Similar frequency of HPV detection was reported by Syrjänen et al. [21] in a Finland population showing an oral prevalence of 15–30% among couples during the 6-year follow-up. A lower but significantly different prevalence of oral HPV in oral lesions was found among Croatian individuals (17%) and Chilean patients (11%) with oral cancer [22,23]. However, in a Brazilian study, it was found the highest prevalence of oral lesions from South America (81%) [24].

Two studies which evaluated oral HPV have been conducted in Buenos Aires, Argentina. These studies found a prevalence rate from 30% to 55%, represented mainly by HPV genotypes 6, 11, and 16 [25,26]. In recent years, the relationship between HPV and the OSCC with the dominance of HPV-16 and -18 genotypes has been reported in Mexico [1]; however, in the present study, the HPV-18 was not detected. In this sense, Syrjänen et al. [3] reported that HPV-16 infection was almost 13 times more frequent in SCC oropharynx and 4 times more frequent in OSCC than in healthy mucous membranes. Similar to our findings, Mravak-Stopetic et al. [22] reported that in a Croatian population, it was found the highest prevalence of high-risk HPV types in PMDs. In addition, we detected genotype 53 in PMDs and OSCC lesions, which is proposed as a type that is probably carcinogenic because of their close phylogenetic relationship with the established carcinogenic types, highlighting the need for continuous control and treatment of these lesions [27].

On the other hand, HPV DNA in asymptomatic infections in which HPV DNA is detectable without any clinical lesion is still a matter of debate. Kreimer et al. [28] reported that asymptomatic oral HPV-16 infection and any HPV type were found in 1.3% and 4.5%, respectively, of the nearly 4.000 subjects included in the review [21]. In a previous local study, HPV in oral mucosa without lesion or injury was not detected [29], maybe because the number of healthy volunteers who were studied was small. Likewise, in a study conducted in the same region among randomly selected healthy subjects, HPV was detected in 3% (13/401) of the cases, and all the identified genotypes were low risk. Most of them exhibited some lesions related to chronic mechanical irritation, and no clinical manifestations suggestive of HPV infection were found in any of the HPV positive subjects [30]. However, oral HPV prevalence range is very wide, and it is influenced by the demographic and ethnic differences of the populations investigated, as well as the different diagnostic procedures [2].

*Chlamydia trachomatis* co-infection is considered to be a possible co-factor that may lead from infection to oncogenesis. Positive associations have been observed between *C. trachomatis* infection and squamous cell carcinoma of the cervix or its precursor lesions in most of the epidemiological studies that detect HPV infection [31]. Indeed, *C. trachomatis* infection can alter the normal structure of epithelial cell junctions, increasing susceptibility to HPV infection both in the genital and oral mucosa. A possible biological explanation for the increased risk for co-infection is that Chlamydia causes local inflammation leading to damage of the epithelial tissue which in turn could become more susceptible to other infections [32].

The frequency of C. *trachomatis* in the oral cavity varies widely among published studies. This variability can be explained by the varied biological samples collected in the different studies (pharyngeal swab, spittle oral wash fluid, and oral lesion samples, etc.), the lack of global standardization techniques used for C. *trachomatis* detection and the diversity of population study groups (men, women, men who have sex with men (MSM), female sex workers, etc.). As a result, it is difficult to make a direct comparison between those studies. Thus, our results show 16% and 18% of *C. trachomatis* DNA positive in oral lesions from women and men, respectively. These frequencies were higher than those in spittle samples among Japanese men (4.2%) [33], in oral wash fluid (3–10%) and in pharyngeal swab (6%) [34]. In addition, in a study conducted in the Netherlands, pharyngeal chlamydia was detected in 1.1% in MSM and in 2.3% among women [35].

However, in a Japanese population of female sex workers, *C. trachomatis* was detected in 44% of pharyngeal swabs and in 61% of oral wash fluid samples [34].

A limitation of this study is that the collected data do not provide us with information concerning the sexual behavior of patients.

With respect to HSV, it has been described that direct contact with lesions or with infected oral or genital secretions during asymptomatic shedding can be a transmission way of the virus [36]. Thus, HSV oral and genital infections may predispose people to HPV infection. Possible mechanisms include HSV infection which enables HPV better access to the basal cell layer, allowing for the establishment of infection [37]. A recent study showed that oral carcinomas co-infected with HPV-16 and HSV-1 had the least favorable outcome, suggesting that HSV-1 infection increases radiation resistance of HPV-16 infected by evasion of apoptosis [38].

Based on this information, we consider it important to study HSV and co-infections with other STIs, which is the aim of the present study. Although a high frequency of HSV in the oral cavity would be expected, in this study the frequency observed was low. This could be due to the fact that lesions directly associated with HSV were not studied, since the biosecurity protocol of dentists does not allow it. In case of visible herpetic injuries, the patients received antiviral treatment and when the treatment is finished, a new sample is taken.

Our study detected HSV DNA in 3% of the cases, represented primarily by HSV-1 (2%) followed by HVS-2 (1%). Most of these were benign lesions and female patients. These results are in agreement with a study carried out in the USA among individuals with HSV-1 and HSV-2 antibody [39] in which isolated oral HSV-2 is less frequent than oral HSV-1 (0.06% and 1%, respectively).

Globally, reports show that the frequency of HSV positivity varies according to the population and type of sample used. Thus, some studies report that HSV-1 was detected in 36% of the oral leukoplakia samples (PMD), and in 52% of the OSCC samples among Sweden patients [40]. Similar to our findings, a previous study in Finland found that only 3% and 2.8% of the asymptomatic female and male were HSV-1 positive in their oral mucosal brush samples, respectively. In addition, this study showed that HSV-1 and HPV co-infection in oral mucosa of young women was rare (0.2%) [38]. In this sense, in our study, we detected only 0.3% of this co-infection (HSV-1 and HPV) and the most frequent co-infection was HSV- *C. trachomatis* (4%).

In the literature, studies of sexually transmitted viruses in lesions of the oral cavity have usually been confined to the search for HPV, so little is known about the presence and co-infection with other infectious agents such as *C. trachomatis* and HSV. The current findings confirm that our knowledge on the variety of infectious agents in the oral cavity is only partial.

In this study, the three studied agents were found in all lesion type regardless of the sex of the patients; therefore, they should be taken into account for diagnostic, treatment purposes, and their epidemiological importance should be considered.

This report is the first contribution to the identification and genotype characterization of HPV in a scenario little studied in our area, and it also contributes to improving our understanding on sexually transmitted infectious agents and their associations with the oral cavity.
